# Medical College and Hospital Notes

**Published:** 1898-06

**Authors:** 


### MEDICAL COLLEGE AND HOSPITAL NOTES.

THE New York University medical college and Bellevue hospital medical college have at last united. It will be remembered that last year a union was attempted between these two famous medical schools and it was defeated on account of differences between the two faculties, then apparently impossible to adjust. Now, however, the amalgamation has been accomplished, an official announcement to that effect having been made by Chancellor MacCracken from the stage of the Metropolitan opera house at the annual commencement of the University medical school, May 18, 1898.

On this occasion 103 graduates received their diplomas, the occasion being enlivened by music from the 7th Regiment Band, and the presence of a house crowded by friends of the institution and of the graduates.

The consolidated institution will be known as the University and Bellevue hospital medical college. The alumni of the two schools, numbering nearly 10,000 graduates, will be placed on the rolls of the university. The two properties, together occupying 225 feet front in East Twenty-sixth street, near First avenue, and costing about $500,000, will be owned by the New York university and used by the united school.

The faculty of the new school will consist of Dr. Edward G. Janeway, dean, and seven professors of the former university medical faculty and twenty-one professors and adjunct professors of the former Bellevue medical faculty, together with such additional professors as may hereafter be appointed. Besides these there are thirty or more lecturers, instructors and assistants.

A MEMORIAL hospital has been presented to the city of Dunkirk by Mrs. F. H. Stevens, of Buffalo, and Mrs. Alfred Solano, of Los Angeles, Cal., joint owners of the handsome residence of the late Horatio Brooks. These charitable women have presented the residence to the Dunkirk Young Men's Association. The building is one of the most imposing structures in that city, having been erected at a cost of $125,000. The main part of the residence will be turned into a memorial hospital in honor of the late Mr. Brooks and the other apartments will be used for a public free library hall and a club room by the Y. M. A.

---

THE Riverside hospital, at 306 Lafayette avenue, Buffalo, founded by and in charge of Dr. Lillian Craig Randall, having outgrown its present proportions will, it is announced, soon be enlarged to meet the increasing demands made upon it for space. When it was established it was located, first, in a small place in Niagara street, near the river, then to larger quarters in Breckenridge street and about two years ago to the two modern buildings in Lafayette avenue. Now these commodious quarters do not suffice for the increased number of patients and the large class of nurses, so it has been decided to add several rooms, including a splendid laboratory, to one of the buildings.

---

A NEW hospital for women at Pittsburg, it is announced, will soon be established. A wealthy, charitably disposed lady—Mrs. Miller—who died recently, bequeathed a valuable tract of land in the East End for the purpose of erecting thereon a hospital for women. She also left to this proposed institution a certain sum of money and made it her residuary legatee. The value of the gift will, it is thought, eventually amount to $300,000. The institution is to be known as the Pittsburg hospital for women. The trustees, among whom are Dr. X. O. Werder and Dr. Simpson, have already applied for a charter.

---

THE Medico-Chirurgical college, of Philadelphia, may now confer D. D. S., Ph. G., and Ph. D. degrees. It is announced that Judge Gordon has dismissed the exceptions of the Philadelphia dental college, which sought to restrain the Medico-Chirurgical college from conferring graduate degrees in dental surgery and pharmacy. The Medico-Chirurgical college's petition to so amend its charter as to comprehend this broadening of the institution's field of usefulness has accordingly been granted.

THE appellate division of the New York supreme court recently reversed an order which granted Professor Charles B. Kelsey a writ of mandamus compelling the directors of the New York Post Graduate Medical school and hospital to rescind a resolution revoking his appointment as a professor of surgery and restoring him to the privilege and all the rights and privileges incident thereto. Justice Barrett wrote the opinion, which held that the by-laws of the corporation authorised the directors to remove a professor at pleasure.

